# Mean platelet volume: a controversial marker of disease activity in Crohn’s disease

**DOI:** 10.1186/2047-783X-17-27

**Published:** 2012-10-12

**Authors:** Song Liu, Jianan Ren, Gang Han, Gefei Wang, Guosheng Gu, Qiuyuan Xia, Jieshou Li

**Affiliations:** 1 Department of Surgery, Jinling Hospital, Medical School of Nanjing University, 305 East Zhongshan Road, Nanjing, 210002, China; 2 Center for the Study of Inflammatory Bowel Disease, Massachusetts General Hospital and Harvard Medical School, 55 Fruit Street, Boston, MA, 02114, USA; 3 Second Affiliated Hospital of Jilin University, Department of General Surgery, General Surgery Center of Jilin University, 218 Ziqiang Road, Changchun, 130041, China; 4 Department of Pathology, Jinling Hospital, Medical School of Nanjing University, 305 East Zhongshan Road, Nanjing, 210002, China

**Keywords:** Crohn’s disease, Mean platelet volume, C-reactive protein, Erythrocyte sedimentation rate, Inflammatory bowel disease

## Abstract

**Background:**

We investigated and compared the capacity of mean platelet volume (MPV) and other inflammatory markers in detecting Crohn’s disease (CD) activity and differentiating CD patients from healthy controls.

**Methods:**

MPV, C-reactive protein (CRP), erythrocyte sedimentation rate (ESR) and white blood cells were measured in 61 CD patients and 50 healthy subjects. Disease activity was assessed by the Crohn’s Disease Activity Index.

**Results:**

A significant decrease in MPV was noted in patients with CD compared with healthy controls (*P* <0.0001), but statistical difference was not found between active and inactive CD groups. In CD, no significant correlation was found between MPV and other inflammatory markers. The overall accuracy of MPV (cutoff: 10.35 fl), CRP (cutoff: 4.85 mg/dl) and ESR (cutoff: 8.5 mm/hour) in differentiating CD patients from healthy controls was 76.6%, 65.8% and 72.1% respectively. The overall accuracy of CRP (cutoff: 4.95 mg/dl) and ESR (cutoff: 16.5 mm/hour) in determination of active CD was 80.3% and 73.8%.

**Conclusions:**

MPV declined in CD patients compared with healthy subjects. MPV had the best accuracy in determination of CD patients and healthy controls. MPV did not show a discriminative value in disease activity.

## Background

The pathogenesis of Crohn’s disease (CD) remains unclear [1,2]. Previous studies suggested that early detection of disease activity could significantly reduce the mortality of CD [3,4]. Non-invasive tests, such as C-reactive protein (CRP), erythrocyte sedimentation rate (ESR) and fecal calprotectin, are therefore being increasingly recognized as important markers for initial diagnosis and disease activity detection.

Recently, several studies have suggested that platelets may be involved in the pathogenesis of CD [5-8]. In addition, mean platelet volume (MPV) has been reported to be influenced in CD [9,10], and has been assumed a potential inflammatory marker and disease activity indicator in several studies [10-12].

However, as these studies involved limited amounts of enrolled patients or did not compare the differential capacity of MPV with previous inflammatory markers, the present study was designed to examine whether MPV would be useful for differentiating CD patients from healthy controls and evaluating CD activity. Furthermore, we analyzed and compared the ability of MPV with other inflammatory markers.

## Methods

### Patients

**Figure 1:**
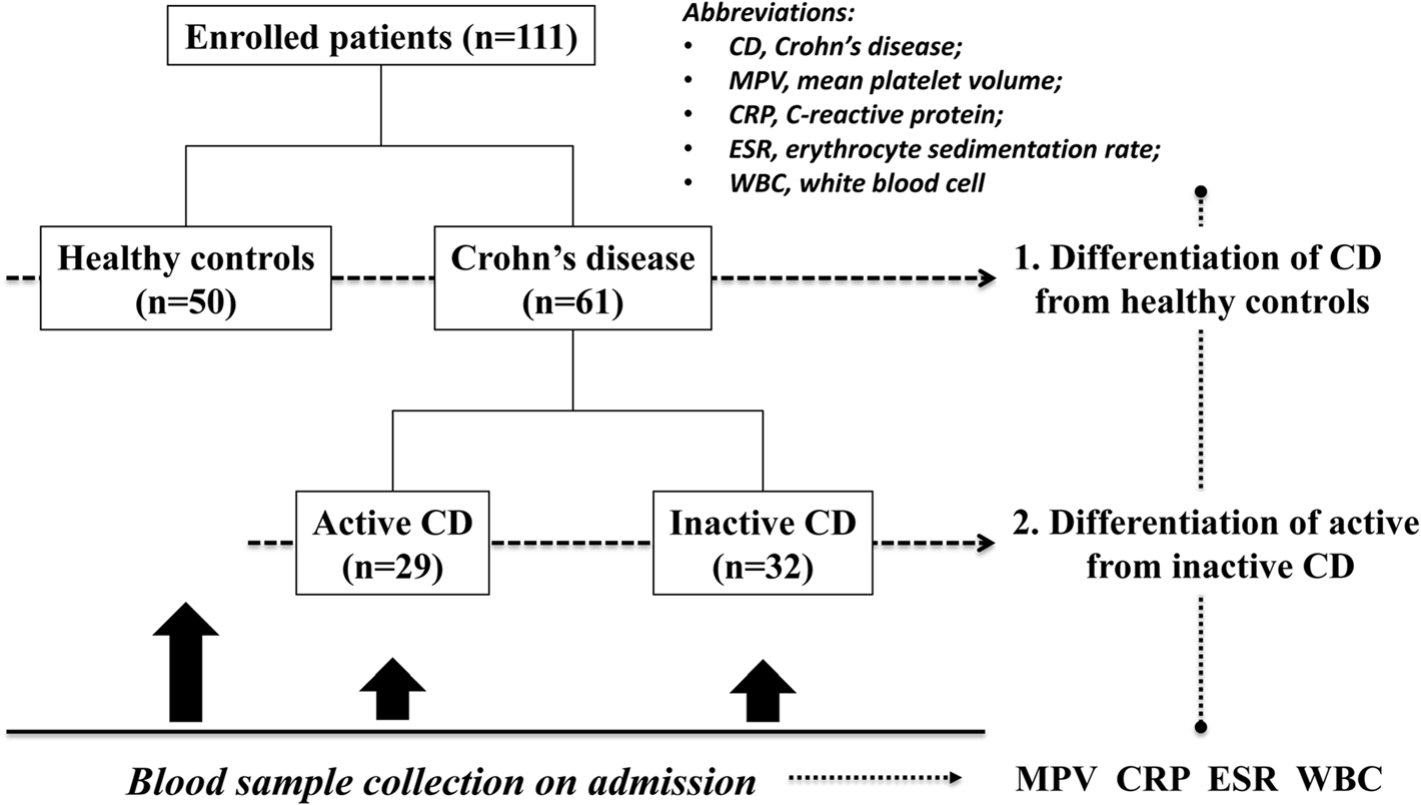
**Study design**. A total of 111 subjects were enrolled in the current study. Fifty healthy controls were differentiated with 61 Crohn’s disease (CD) patients using mean platelet volume (MPV), C-reactive protein (CRP), erythrocyte sedimentation rate (ESR) and white blood cells (WBC). Furthermore, the 61 CD patients were divided into active (*n* = 29) and inactive (*n* = 32) groups and distinguished using the same inflammatory biomarkers. All blood sample collections were obtained on admission (before any medication or procedure).

We prospectively collected 61 CD patients and 50 healthy subjects between March 2010 and September 2011 (Figure 1). The diagnostic criteria of CD were mainly composed from standard clinical, radiological, endoscopic and histopathologic findings.

The exclusion criteria were acute or chronic infection, hypertension, endocrinological disorder, hematological disease, heart failure, hepatic and renal disorder, cancer and peripheral vascular disease [13]. None of the enrolled subjects had received anticoagulant medications, NSAIDs or contraceptives.

### Laboratory parameters

Full blood count (including MPV and white blood cells (WBC)), CRP and ESR were performed on admission (before prescribing any medications).

For MPV measurement in all enrolled patients, the blood samples were anticoagulated by ethylenediamine tetraacetic acid with wortmanin and tyrphostin [14], and then treated with rapid processing (within <4 hours) [14] and the same storage temperature [13,15]. For other parameters, the measurement and process were carried out according to standard laboratory practice.

To calculate the body mass index, height and weight were recorded on admission for each individual. For CD patients, the disease activity was defined according to the Crohn’s Disease Activity Index (CDAI) score. Patients were further divided into an active CD group (CDAI >150) and an inactive CD group (CDAI <150) (Figure 1).

### Statistical analysis

Statistical analysis was performed using GraphPad Prism Software (version 5.01; GraphPad, San Diego, CA, USA). All analyses were two-tailed and differences were considered statistically significant when *P* <0.05. For continuous variables, the mean and standard error of the mean were calculated; Students’ *t* test was used to compare variance between groups. For categorical variables, percentages were provided and the chi-squared test was used. Pearson analysis was used to calculate the correlation between MPV and other inflammatory markers. Receiver operating characteristic (ROC) curve analysis was performed to identify optimal cutoff values for MPV and other inflammatory markers. The overall accuracy was also calculated by additional true-positive and true-negative test results divided by all tests: (a + d) / (a + b + c + d).

### Ethical consideration

This study was approved by the Ethics Committee of Jinling Hospital, and a written informed consent was obtained from each enrolled participant.

## Results

### Differentiation of Crohn’s disease patients and healthy controls

The demographic features of CD patients and healthy controls are shown in Table 1. The distributions of age, gender, smoking habit and body mass index were not statistically significant between groups.

**Table 1:** Demographics of patients and controls

	**Crohn’s disease (*n *= 61)**	**Control group (*n *= 50)**	***P *value**
Age (years)	32.4 ± 1.59	34.0 ±1.44	0.133
Male (%)	40 (65.6%)	35 (70.0%)	0.686
Smoking	15 (24.6%)	11 (22.0%)	0.709
Body mass index (kg/m^2^)	18.4 ± 0.526	19.3 ± 0.507	0.427
Active disease	29 (47.5%)	–	–
Age (%)			
A1 (≤16 years)	9 (14.7%)	–	–
A2 (17–40 years)	37 (60.7%)	–	–
A3 (>40 years)	15 (24.6%)	–	–
Disease location (%)			
L1 (ileal)	33 (54.1%)	–	–
L2 (colonic)	10 (16.4%)	–	–
L3 (ileocolonic)	18 (29.5%)	–	–
+ L4 (upper gastrointestinal tract)	3 (4.92%)	–	–
Disease behavior (%)			
B1 (inflammatory)	9 (14.7%)	–	–
B2 (stricturing)	32 (52.5%)	–	–
B3 (penetrating)	20 (32.8%)	–	–
+ P (perianal)	3 (4.92%)	–	–

Table 2 demonstrates comparisons of all inflammatory markers, including MPV, CRP, ESR and WBC. A significant decline in MPV was noted in patients with CD compared with healthy controls (9.55 ± 0.168 fl vs. 11.1 ± 0.160 fl, *P* <0.0001). Meanwhile, CRP (13.4 ± 2.43 mg/dl vs. 2.89 ± 0.547 mg/dl, *P* <0.0001) and ESR (19.1 ± 2.15 mm/hour vs. 6.60 ± 0.431 mm/hour, *P* <0.0001) were statistically higher in the CD group than those in the control group. However, WBC appeared to be similar in both groups (6.83 ± 0.377×10^9^/l vs. 6.90 ± 0.342×10^9^/l, *P* = 0.8881).

**Table 2:** Comparison of MPV and other inflammatory markers between Crohn’s disease and control groups

	**Crohn’s disease (*n *= 61)**	**Control group (*n *= 50)**	***P *value**
MPV (fl)	9.55 ± 0.168	11.1 ± 0.160	**<0.0001**
CRP (mg/dl)	13.4 ± 2.43	2.89 ± 0.547	**<0.0001**
ESR (mm/hour)	19.1 ± 2.15	6.60 ± 0.431	**<0.0001**
WBC (×10^9^/l)	6.83 ± 0.377	6.90 ± 0.342	0.8881

CRP, C-reactive protein; ESR, erythrocyte sedimentation rate; MPV, mean platelet volume; WBC, white blood cells.

We further investigated the ability of MPV and other markers in differentiating CD patients from healthy controls. As shown in Table 3, the optimal cutoff value for MPV was 10.35 fl, with sensitivity and specificity of 78.7% and 74.0% respectively (area under the curve (AUC): 0.8303). The overall accuracy of MPV in detecting CD patients was 76.6%.

**Table 3:** Accuracy and ROC analyses of MPV and other inflammatory markers in differentiate patients and controls

	**AUC**	**Sensitivity(%)**	**Specificity(%)**	**Overall accuracy(%)**
MPV (cutoff: 10.35)	**0.8303**	78.7	74.0	76.6
CRP (cutoff: 4.85)	**0.6849**	52.5	82.0	65.8
ESR (cutoff: 8.5)	**0.7834**	68.9	76.0	72.1
WBC (cutoff: 4.35)	0.5234	18.0	92	51.4

AUC, area under the curve; CRP, C-reactive protein; ESR, erythrocyte sedimentation rate; MPV, mean platelet volume; ROC, receiver operating characteristic; WBC, white blood cells.

Moreover, ROC analysis also suggested 4.85 mg/dl and 8.5 mm/hour as optimal cutoff points for CRP (sensitivity: 52.5%, specificity: 82%, AUC: 0.6849) and ESR (sensitivity: 68.9%, specificity: 76.0%, AUC: 0.7834) respectively (Figure 1).

### Differentiation of active and inactive Crohn’s disease

According to CDAI scores, patients with CD were assigned into active and inactive groups. Notably, MPV was similar between active and inactive CD patients (9.52 ± 0.223 fl vs. 9.58 ± 0.251 fl, *P* = 0.8423) (Table 4). However, CRP (21.8 ± 4.21 mg/dl vs. 5.75 ± 1.81 mg/dl, *P* = 0.0012) and ESR (26.4 ± 3.26 mm/hour vs. 12.5 ± 2.33 mm/hour, *P* = 0.0011) of active CD patients were significantly higher than those of inactive CD patients.

**Table 4:** Comparison of MPV and other inflammatory markers in patients with and without active disease

	**Active CD (*n *= 29)**	**Inactive CD (*n *= 32)**	***P *value**
MPV (fl)	9.52 ± 0.223	9.58 ± 0.251	0.8423
CRP (mg/dl)	21.8 ± 4.21	5.75 ± 1.81	**0.0012**
ESR (mm/hour)	26.4 ± 3.26	12.5 ± 2.33	**0.0011**
WBC (×10^9^/l)	6.42 ± 0.413	7.19 ± 0.613	0.3047

CD, Crohn’s disease; CRP, C-reactive protein; ESR, erythrocyte sedimentation rate; MPV, mean platelet volume; WBC, white blood cells.

Spearman correlation analysis suggested that MPV did not correlate with CRP (*r* = −0.022, *P* = 0.8671), ESR (*r* = −0.059, *P* = 0.6518) or WBC (*r* = −0.1549, *P* = 0.2332) in patients with CD (Table 5).

**Table 5:** Correlation between MPV and other inflammatory markers in Crohn’s disease

**MPV**	***r *value**	***P *value**
CRP	−0.022	0.8671
ESR	−0.059	0.6518
WBC	−0.1549	0.2332

CRP, C-reactive protein; ESR, erythrocyte sedimentation rate; MPV, mean platelet volume; WBC, white blood cells.

**Figure 2:**
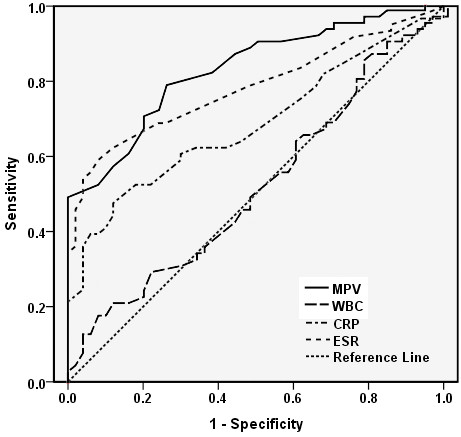
**Mean platelet volume and other inflammatory markers in differentiate Crohn**’**s disease patients and controls**. Receiver operating characteristic (ROC) curves. The optimal cutoff value for mean platelet volume (MPV) was 10.35 fl (sensitivity: 78.7%, specificity: 74.0%, area under the curve: 0.8303), with 76.6% overall accuracy.

**Figure 3:**
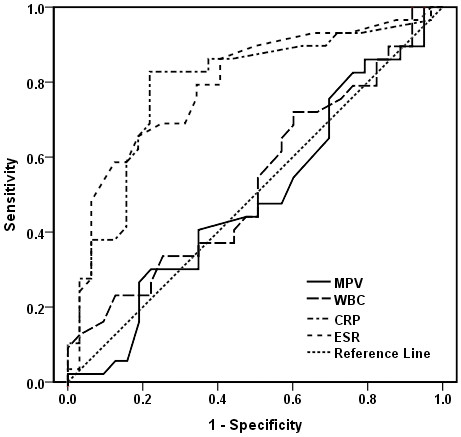
**Mean platelet volume and other inflammatory markers in differentiate active and inactive Crohn**’**s disease**. Receiver operating characteristic (ROC) curves. The optimal cutoff levels for C-reactive protein (CRP) and erythrocyte sedimentation rate (ESR) were 4.95 mg/dl (sensitivity: 82.8%, specificity: 78.1%, area under the curve (AUC): 0.7877) and 16.5 mm/hour (sensitivity: 65.5%, specificity: 81.3%, AUC: 0.7909), with 80.3% and 73.8% overall accuracy respectively. MPV did not show a statistically discriminative value in differentiating active from inactive Crohn’s disease (CD) (AUC: 0.5043, overall accuracy: 55.7%)

We further performed ROC analysis to investigate the capacity of all inflammatory markers in differentiating active from inactive CD (Table 6 and Figure 2). The optimal cutoff levels for CRP and ESR were 4.95 mg/dl (sensitivity: 82.8%, specificity: 78.1%, AUC: 0.7877) and 16.5 mm/hour (sensitivity: 65.5%, specificity: 81.3%, AUC: 0.7909), with 80.3% and 73.8% of overall accuracy respectively (Figure 3). However, MPV did not show a statistically discriminative value in differentiate active from inactive CD (AUC: 0.5043, overall accuracy: 55.7%).

**Table 6:** Overall accuracy and ROC analyses of MPV and other inflammatory markers in differentiating Crohn’s disease

	**AUC**	**Sensitivity (%)**	**Specificity (%)**	**Overall accuracy (%)**
MPV (cutoff: 8.80)	0.5043	31.0	78.1	55.7
CRP (cutoff: 4.95)	**0.7877**	82.8	78.1	80.3
ESR (cutoff: 16.5)	**0.7909**	65.5	81.3	73.8
WBC (cutoff: 7.20)	0.5474	72.4	40.6	55.7

CRP, C-reactive protein; ESR, erythrocyte sedimentation rate; MPV, mean platelet volume; ROC, receiver operating characteristic; WBC, white blood cells.

## Discussion

In the present study we have demonstrated that the MPV level was significantly lower in CD patients than that in healthy participants. We also illustrated that MPV was an accurate marker in distinguishing CD patients from healthy controls. However, the MPV level was statistically similar between active and inactive CD patients, leading to a failure of detecting patients with or without active disease.

Meanwhile, we demonstrated that CRP and ESR were both higher in CD patients compared with healthy controls, and higher in active compared with inactive CD patients. Even though the overall accuracy of CRP and ESRwas lower than that of MPV in detecting CD patients, they were still effective in determination of active CD patients.

Moreover, we found that MPV was not correlated with CRP, ESR or WBC in patients with CD. WBC was not an effective indicator in distinguishing CD patients from healthy subjects and active from inactive CD patients.

CD is characterized by chronic, transmural intestinal inflammation in which periods of remission with variable length are interrupted by relapse episodes. Previous studies have demonstrated that appropriate and effective therapy could significantly control symptoms, maintain remission, prevent relapse, improve quality of life and reduce mortality [3,16]. The early determination of diagnosis and detection of disease activity are therefore essential for tailoring therapy [17].

As invasive techniques, including endoscopic, radiological and histopathologic methods, are routinely used for diagnostic decision and disease activity supervision, an ideal non-invasive test is increasingly expected for initial diagnosis and identification of disease activity [18].

CRP is a valuable inflammatory marker in inflammatory bowel disease (IBD), especially CD [17,19]. Prior studies suggested a sensitivity range for uncovering IBD between 50 and 60% [20,21]. In our study, CRP displayed a high accuracy for differentiating CD patients from healthy controls and for distinguishing patients with and without active disease (65.8% and 80.3% respectively).

ESR and WBC are both nonspecific markers of inflammation. Although they could adjust to and reflect the severity of inflammation, low sensitivity and specificity of gastrointestinal inflammatory status were reported in previous studies [19,22]. However, in the present study, ESR performed as a notable marker in the detection of CD patients (overall accuracy: 72.1%) and in distinguishing active from inactive CD (overall accuracy: 73.8%).

Recently, a series of stool tests, such as fecal lactoferrin, calprotectin and elastase, were investigated as novel inflammatory markers. Even though they may be superior to CRP or ESR with higher sensitivity and specificity in detecting gastrointestinal inflammation [17,23,24], they are not specific markers for IBD; and they are inconvenient and unpleasant for stool sampling.

The link between MPV and inflammation has been well investigated in the literature [13,25]. Previous series of case–control and cohort studies have discovered several important confounders of MPV. Smoking and exposure to nicotine were key factors that would significantly influence platelet morphology and size, and could be confounded by sex, age and duration of smoking [13]. In the current study, the age, sex and smoking histories were statistically similar between healthy controls and patients with CD (Table 1). However, the duration of smoking was not registered and calculated in this study, leading to a difficulty in excluding the influence of duration of smoking on MPV. Nevertheless, as the percentage of participants with a current or previous smoking history was <25% in both healthy control (22.0%) and CD (24.6%) groups, the influence of duration of smoking on MPV was dramatically restricted. Hypertension [13,26] and diabetes [27,28] were confirmed closely correlated with large platelet size, respectively. To prevent their influence on MPV, we excluded patients with hypertension, heart failure, hepatic and renal disorder, vascular disease, endocrinological disorder and hematological disease. Obesity was also found to be associated with elevated MPV [13,29]. However, the normal and similarity of body mass index in both healthy controls and CD patients eliminated the potential influence of obesity on MPV.

Platelets, which are frequently complicated with thromboembolic risks, may play a crucial role in the pathogenesis of IBD [30-33]. The statistically significant declined MPV in CD patients observed in the present study is in accordance with findings of prior studies [9,10], suggesting that MPV may be helpful in clinical practice [34,35].

To our knowledge, prior studies have never analyzed and reported a positive result of MPV in differentiating CD patients from healthy volunteers using ROC analysis. Our study, for the first time, discovered a preliminary but inspiring finding that MPV could be used as a good biomarker in distinguishing CD from healthy controls (Table 3). We are aware that our finding is not sufficient for employing MPV solely in this differential diagnosis, but our finding could provide a suggestion for clinical physicians to pay attention to the value of MPV and take it into account when making decisions of differential diagnosis.

The similar MPV between active and inactive CD patients observed in our study is not in agreement with those in previous studies. The absence of any correlation between MPV and other inflammatory markers in our study supported the hypothesis that MPV was not in a close relationship with CD activity. In the studies by Jaremo and Sandberg-Gertzen [10] and Kapsoritakis and colleagues [12], an activity-dependent diversity of MPV was reported. The reason for this discrepancy is unclear; we assume that the amount of enrolled patients in these studies was limited, and large-sample studies are expected to investigate the real diversity between active and inactive CD patients.

Our study raised several interesting and meaningful controversies that are worthy of discussions and further investigations.

Firstly, we raised controversy about the correlation between MPV and other inflammatory biomarkers in CD.

Although the issue of MPV shifts in inflammatory disorders has been extensively covered in recent publications, studies in the current literature investigating the association of MPV and CD are still limited. Among them, studies discussing the correlation between MPV and other regular inflammatory biomarkers (including CRP, ESR and WBC) in CD are even fewer.

The only and most worthy-of-mention study was published in 2001. Kapsoritakis and colleagues collected data for 66 patients with CD and 38 healthy volunteers, and found a negative correlation of MPV with CRP, ESR and WBC [12]. However, they did not analyze and compare the cutoff point, sensitivity, specificity and overall accuracy of MPV and other biomarkers.

Our results are not in accordance with their findings. As no statistical correlation between MPV and other inflammatory biomarkers was confirmed in our study, an obvious and meaningful controversy appeared. While the sample size was similar between our study (*n* = 61) and that of Kapsoritakis and colleagues (*n* = 66), further studies are expected to uncover the real relationship between MPV and other biomarkers. This confirmation of relationship is pivotal in determining the role of MPV in the inflammatory course in CD.

Secondly, we raised a controversy about the decisive value of MPV in the determination of activity in CD.

The similarity of MPV between active and inactive CD in our study is in contrast with several previous remarkable studies. Jaremo and Sandberg-Gertzen collected data for 18 ulcerative colitis patients, nine CD patients and 18 healthy volunteers, and reported an association of lower MPVs with active IBD [10]. However, this excessive small sample size of CD (nine patients) significantly hampered the value of their conclusions. Yuksel and colleagues recruited data for 61 ulcerative colitis patients and 27 healthy controls, and suggested that decreased MPV may be an indicator for increased disease activity in patients with ulcerative colitis [35]*.* However, they did not collect data for CD patients or provide any information regarding MPV in indicating CD activity.

Douda and colleagues collected data for 56 CD patients and found that decreased MPV is an independent laboratory marker of clinical disease activity [36]. However, MPV’s predictive value is inferior compared with the total platelet count, CRP and CDAI. The latter finding is partly in accordance with our findings. We also discovered that MPV was inferior to CRP and ESR in determination of activity in CD (see Table 6), but it is still a challenge for clinical physicians to find more evidence to prove a definite association between MPV and disease activity.

There has been sufficient evidence derived from numerous prospective and retrospective studies suggesting MPV is a key predictor of thrombotic events in various disorders [13], including cardiovascular disease [37], cerebrovascular disease [38,39], venous thromboembolism [40] and other disorders [41,42].

In IBD, low-sized platelet with an increase of platelet count has been a typical manifestation for both CD and ulcerative colitis [13]. Meanwhile, both a prothrombotic condition and a hypercoagulable state are established features of IBD [43]. Microaggregates and microinfarction of mesenteric vessels unveiled a potential role of platelet as an inflammatory cell in the pathogenesis of CD [8,13]. Wakefield and colleagues demonstrated in their study a consecutive process of microinfarction in mesenteric vasculature [44]. This process was initiated by vascular injury, followed by focal arteritis, fibrin deposition and arterial occlusion at muscularis mucosa. Subsequently, tissue infarction and mucosal ulceration were assumed to be a possible chain of events in the pathogenesis of CD [45]. Notably, the above course of events depended on several key factors – including glycoprotein IIb/IIIa, which is a platelet surface glycoprotein, and platelet surface template for factors V and VIII, which were previously known as platelet factor 3 [46]. Platelets were therefore proposed to be involved, at least partly, in the formation of microinfarction and the pathogenesis of CD [45].

An increased activation of platelet was confirmed by the expression of surface activation markers including P-selectin, GP53 and β-thromboglobulin [43,47]. Moreover, various factors (such as IL-3, IL-6 and thrombopoietin) were responsible for the platelet activation and participated in the stimulation of thrombopoiesis [48]. The reduced size of platelets in CD suggested a possible mechanism in which large activated platelets were consumed or sequestrated in the intestinal vasculature [49].

We are aware of limitations to our study. First, this is a single-center study, leading to a potential selection bias. Second, as only 61 CD cases and 50 healthy subjects were enrolled in our study, the sample size might be too small to detect the real diversity of MPV between active and inactive CD patients.

## Conclusions

Our study demonstrated a decline of MPV in CD patients compared with healthy controls. We also compared MPV with other inflammatory markers, including CRP, ESR and WBC, and provided the discriminative talent of MPV and recommended MPV as the best marker for differentiating CD patients from healthy subjects. Finally, we suggested that it should be cautious to use MPV as a marker in determination of CD activity. Large multicenter studies are expected to resolve the controversy.

## Abbreviations

AUC: area under the curve; CD: Crohn’s disease; CDAI: Crohn’s Disease Activity Index; CRP: C-reactive protein; ESR: erythrocyte sedimentation rate; IBD: inflammatory bowel disease; IL: interleukin; MPV: mean platelet volume; NSAID: nonsteroidal anti-inflammatory drug; ROC: receiver operating characteristic; WBC: white blood cells.

## Competing interests

The authors declare that they have no competing interests.

## Authors’ contributions

SL and JR participated in the design of the study. SL and GH performed the statistical analysis. GW and GG collected the data and helped to draft the manuscript. JL conceived of the study, and participated in its design and coordination. SL and QX drafted the manuscript. All authors read and approved the final manuscript.
